# Regulation of Metabolic Pathways by MarR Family Transcription Factors

**DOI:** 10.1016/j.csbj.2017.06.001

**Published:** 2017-06-16

**Authors:** Anne Grove

**Affiliations:** Department of Biological Sciences, Louisiana State University, Baton Rouge, LA 70803, USA

**Keywords:** Biosensor, Gene expression, HucR, Ligand binding, Lignin catabolism

## Abstract

Bacteria have evolved sophisticated mechanisms for regulation of metabolic pathways. Such regulatory circuits ensure that anabolic pathways remain repressed unless final products are in short supply and that catabolic enzymes are not produced in absence of their substrates. The precisely tuned gene activity underlying such circuits is in the purview of transcription factors that may bind pathway intermediates, which in turn modulate transcription factor function and therefore gene expression. This review focuses on the role of ligand-responsive MarR family transcription factors in controlling expression of genes encoding metabolic enzymes and the mechanisms by which such control is exerted. Prospects for exploiting these transcription factors for optimization of gene expression for metabolic engineering and for the development of biosensors are considered.

## 1 Introduction

Bacterial ligand-responsive transcription factors may sense environmental agents or cellular metabolites to effect differential regulation of target genes, a regulation that typically occurs at the level of transcription initiation. In many cases, ligand- and DNA-binding functions reside in the same protein, either within a single domain as seen in members of the multiple antibiotic resistance regulator (MarR) protein family [1], or separated in distinct domains that communicate via a linker region as exemplified by the lactose repressor (LacI) family regulators [2]. A common scaffold includes the helix-turn-helix DNA-binding domain combined with an allosteric domain to which the metabolic intermediate or exogenous compound associates. Many such transcription factors exist as homodimers or even higher order oligomeric assemblies, and they are frequently autoregulatory. Cognate DNA sites – often palindromic sequences – usually reside within gene promoters, allowing bound transcription factor to activate or repress gene activity. The regulatory function is dictated by the precise location of the binding site, with repressors typically interfering with binding of RNA polymerase or impeding its movement on DNA whereas activators generally bind further upstream to assist in polymerase recruitment. In addition to direct interaction with RNA polymerase, some transcription factors may modulate gene activity by altering promoter DNA topology [3], [4], [5].

**Fig. 1:**
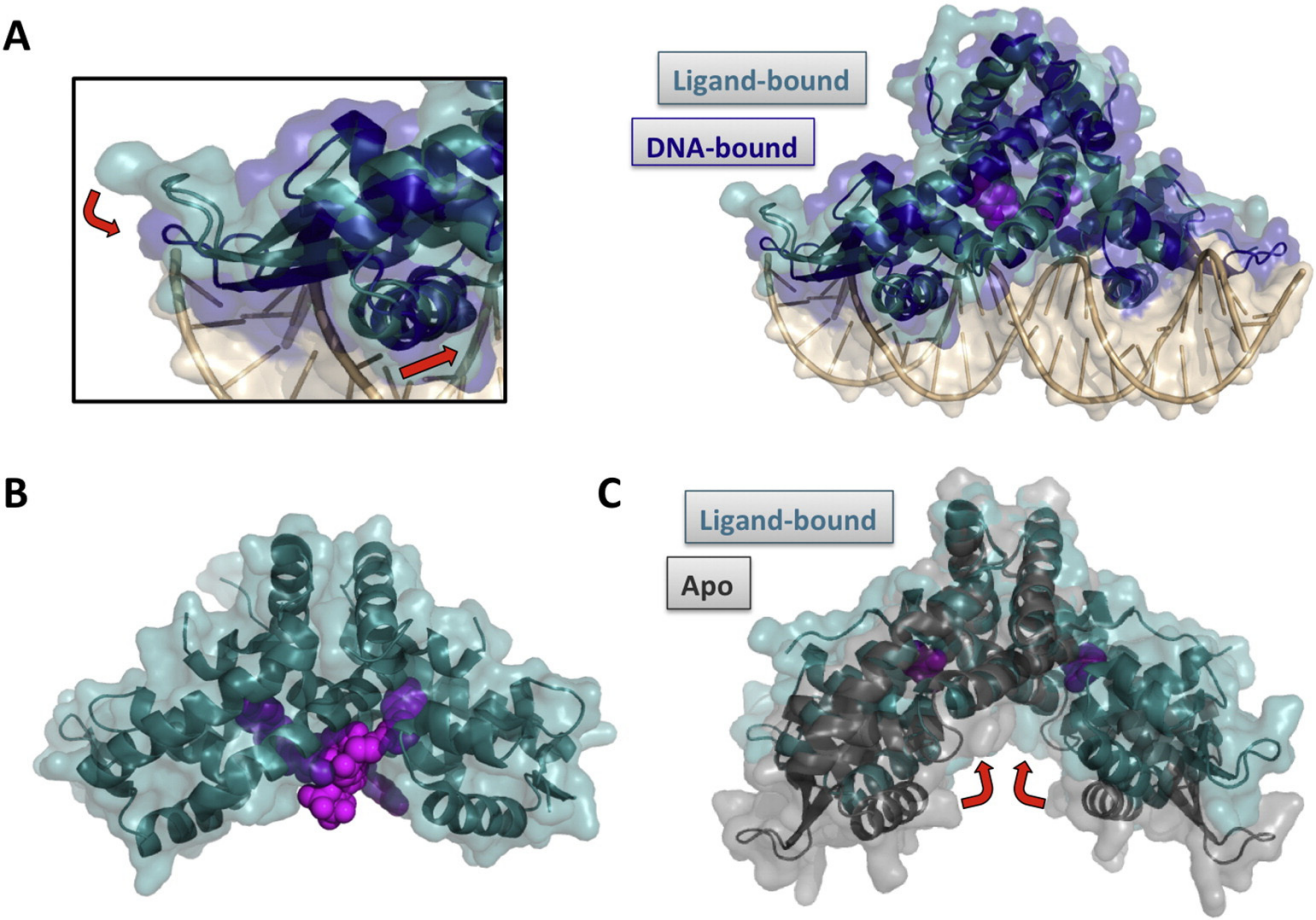
Consequences of ligand binding to MarR homologs. A. DNA-bound HcaR (5BMZ; blue) overlayed with ligand-bound HcaR (4RGX; teal) [12]. Ligand-binding does not induce significant conformational changes in HcaR and is proposed to stabilize apo-HcaR (not shown). DNA is shown in tan, and the ligands (protocatechuate) are depicted in magenta. Each ligand binds in a crevice between DNA-binding and dimerization regions. The left panel is a close-up of the DNA-binding wHTH motif, showing the adjustment of the recognition helix and the shift of the wing towards the minor groove that is induced on DNA binding (red arrows). B. Structure of CouR in complex with coumaroyl-CoA (5CYV). CouR monomers are shown in light and dark teal and the two ligands in light and dark magenta. CoA moieties are proposed to interfere sterically and electrostatically with DNA binding [24]. C. Structures of apo-PcaV (4G9Y; gray) and protocatechuate-bound PcaV (4FHT; teal; ligand in magenta), superposed via dimerization regions. A rigid-body movement of wHTH motifs is induced on ligand binding (highlighted by red arrows). Figure generated with PyMol. (For interpretation of the references to color in this figure legend, the reader is referred to the web version of this article.)

**Fig. 2:**
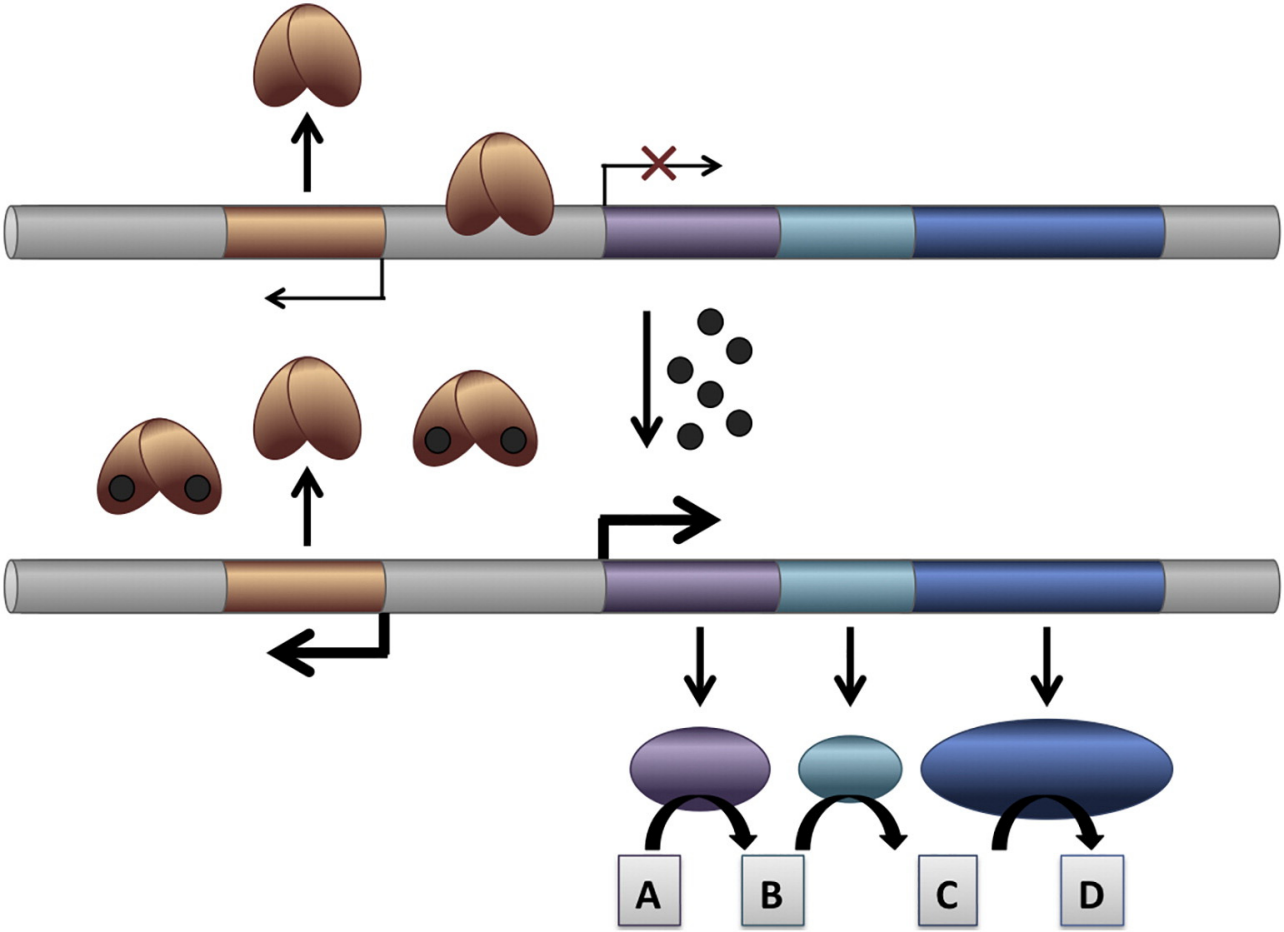
Differential gene expression controlled by ligand-responsive transcription factor. The gene encoding transcription factor (copper) may be divergent to operon encoding enzymes that participate in specific metabolic pathway in which compound A is converted to D via B and C (blue). DNA-bound transcription factor represses expression of the divergent genes (top), whereas binding of ligand (black) induces a conformational change in the protein that causes it to release from the DNA (bottom). Ligands that lead to derepression are frequently early pathway intermediates. (For interpretation of the references to color in this figure legend, the reader is referred to the web version of this article.)

A change in the conformation or flexibility of the transcription factor is elicited upon ligand binding, and this results in altered interaction with cognate DNA and therefore a change in gene expression (Fig. 1). Such allosteric modulation of DNA binding may manifest as either increased or attenuated DNA binding, thereby allowing the ligand to flip a molecular on–off switch or to adjust a tunable dimmer-switch-like system (Fig. 2). As sensors of environmental cues, many transcription factors that follow this paradigm control production of virulence factors in response to host-derived signals. Others control metabolic processes in response to accumulation of nutrients or specific pathway intermediates [1], [6], [7], [8].

The ability to sense and metabolize specific nutrients confers a fitness advantage, as it avoids costly synthesis of unnecessary enzymes and allows utilization of resources that competing species may not be able to catabolize. In addition, genes encoding enzymes that participate in a specific pathway are often encoded together in an operon, a genome organization that allows concerted regulation of all genes. While metabolic pathways are often conserved, regulatory networks vary between species. This review highlights examples of ligand-responsive MarR family transcription factors that respond to specific pathway intermediates to effect differential gene expression; a particular focus is on MarRs that respond to lignin-derived aromatics, as high resolution structural analyses have been reported that illustrate distinct mechanisms by which binding of structurally related ligands may alter association with cognate DNA. Advances towards optimizing or (re)designing MarR protein function for metabolic engineering or generation of biosensors are discussed.

## 2 The MarR Protein Family

Named for the *Escherichia coli* Multiple Antibiotic Resistance Regulator, MarR family transcription factors are ubiquitous in the bacterial kingdom [1], [8]. Generally, bacterial species characterized by a large genome size and a complex lifestyle that includes both free-living and parasitic or symbiotic stages encode a greater number of transcription factors, including MarRs [9]. In contrast, obligate parasitic species that feature reduced genome sizes or species with restricted niches encode few regulatory networks. For example, a search of the genome of the specialized gastric pathogen *Helicobacter pylori* does not uncover any MarR homologs whereas the human pathogen *Staphylococcus aureus*, which can infect multiple ecological niches within the host environment, encodes 18 [10]. A search of the > 40,000 bacterial genomes available through Ensembl Bacteria for “MarR” suggests an average of ~ 7 MarR paralogs per genome. As sensors of changing environments, MarR proteins are particularly well suited to control expression of virulence genes, as exemplified by proteins such as SlyA and PecS that are master regulators of virulence genes in either human or plant pathogens [11]. Control of genes encoding antibiotic efflux pumps is another well-documented role of MarR proteins [8]. Given the vast number of predicted MarR homologs, however, only a small proportion has been experimentally characterized.

MarR proteins exist as obligate homodimers in which each monomer contributes a winged helix-turn-helix (wHTH) DNA-binding motif. Recognition helices from each DNA-binding motif bind palindromic DNA sequences in consecutive major grooves, with residues in the wing contacting the adjacent minor grooves (Fig. 1A) [12]. DNA- and sensor-binding regions reside within one protein domain, and communication between these protein regions depends on the dynamics of the dimer interface [13], [14], [15]. While some MarR proteins are regulated by cysteine oxidation, including the eponymous *E. coli* MarR [16], most bind small molecule ligands, often phenolic compounds. Many MarR proteins share a ligand-binding “hot-spot” at the interface between DNA-binding and dimerization interfaces (Fig. 1), whereas others have evolved different ligand-binding modes [1].

## 3 Regulation of Metabolic Pathways

Bacteria continuously monitor their environment and cellular metabolic state and modify their gene expression patterns in response to perceived cues. The ensuing changes in gene activity are the result of complex transcriptional networks that may include global regulators that control a large number of transcription units (such as the cAMP Receptor Protein, CRP, that functions in control of genes whose products are involved in carbon utilization) as well as more specific transcription factors that are dedicated to particular genes [3]. Carbon sources are particularly important in terms of cell proliferation and generation of energy. Glucose is typically the preferred carbon source, and its presence often prevents the utilization of alternative carbon sources by the process of Carbon Catabolite Repression [17]. In addition, the presence of a specific alternate carbon source is required for expression of genes encoding the corresponding catabolic enzymes.

### 3.1 Catabolism of Lignin Derivatives

Mineralization of lignin-derived aromatic compounds by soil bacteria is a key step in the terrestrial carbon cycle, and several bacterial species have been characterized that can utilize such compounds as carbon and energy sources [18], [19]. These aromatics may be degraded aerobically to common intermediates such as catechol, protocatechuate (3,4-dihydroxy benzoate), or gentisate and then converted to intermediates in the citric acid cycle, or they may be processed anaerobically to benzoyl-CoA and phenylacetyl-CoA and subsequently channeled into central metabolism [20]. Transcription factors belonging to several different protein families have been characterized that control expression of genes encoding the corresponding catabolic enzymes.

One example is the MarR protein HcaR from *Acinetobacter baylyi* ADP1, which controls expression of the *hca* operon that encodes proteins involved in catabolism of the *p*-hydroxycinnamic acid derivatives caffeate, *p*-coumarate, and ferulate. Hydroxycinnamates are constituents of lignin and other plant components, and they are precursors in the synthesis of flavonoids. For *A. baylyi* ADP1 HcaR, the inducing metabolites were reported to be the hydroxycinnamate-CoA thioesters [21]. The crystal structure of HcaR bound to inducing ligand (protocatechuate in the structure shown; Fig. 1A) illustrates a common paradigm among MarR family proteins, with the ligand binding in a crevice between DNA-binding and dimerization regions of the protein (right panel). While apo-HcaR and ligand-bound HcaR are structurally very similar, DNA-binding is associated with conformational changes in which the wHTH motif is adjusted and the wing is shifted ~ 6 Å towards the DNA minor groove (Fig. 1A; left panel) [12]. The interpretation is that ligand stabilizes a protein conformation that is incompatible with DNA binding, precluding required conformational changes. This mode of ligand-mediated control of DNA binding has also been proposed for other MarR proteins, for example *Neisseria* NadR, which controls expression of an adhesin that mediates binding to human cells [22]. Similarly, molecular dynamics analyses of *Pseudomonas aeruginosa* MexR, which regulates expression of genes encoding the MexAB-OprM efflux pump, suggested that a flexible DNA-binding state is reached only transiently and that ligand-binding shifts the conformational ensemble towards a less flexible conformation that cannot bind DNA [23].

*Rhodococcus jostii* RHA1 can also grow on *p*-hydroxycinnamate derivatives such as *p*-coumarate. Catabolism requires expression of the *cou* genes, which are under control of CouR. CouR acts as a repressor, and gene expression is induced on binding of the inducer *p*-coumaroyl-CoA, but not *p*-coumarate. Structural analysis revealed binding of two ligand molecules per protein dimer, with no significant structural differences on ligand binding compared to apo-CouR. However, while the phenolic ligand moieties occupy equivalent hydrophobic pockets between DNA-binding and dimerization regions of the protein, the CoA parts extend down and are predicted to interfere sterically with DNA binding as well as impose a charge repulsion (Fig. 1B) [24].

In *Sphingobium* sp. strain SYK-6, FerC controls expression of genes encoding enzymes required for degradation of the lignin-derivative ferulate. Binding of FerC to its operator site was inhibited by the CoA-thioester of ferulate and other hydroxycinnamoyl-CoAs [25]. Similarly, inhibition of DNA binding by *Rhodopseudomonas palustris* CouR occurs on binding of coumaroyl-CoA [26]. Thus, induction by CoA-thiester derivatives is emerging as a shared property of MarR proteins involved in control of catabolism of lignin derivatives, yet *A. baylyi* ADP1 HcaR appears to bind the unesterified ligand to stabilize a conformation that is unfavorable for DNA binding. It would be of interest to complete these structural comparisons by determining if the failure to induce *R. jostii* RHA1 CouR by *p*-coumarate is reflected in a ligand-bound conformation that is compatible with DNA binding.

In the β-ketoadipate pathway, catechol and protocatechuate are converted into β-ketoadipate and subsequently into citric acid cycle intermediates. The diversity of transcriptional regulators involved in control of genes encoding enzymes of the β-ketoadipate pathway illustrates how different bacterial species have adapted to aromatic compound degradation as regulators belonging to LysR, IclR, and MarR families have been described [27], [28]. In *Streptomyces coelicolor*, the MarR family regulator PcaV represses expression of the *pca* operon that encodes enzymes required for conversion of protocatechuate to acetyl-CoA and succinyl-CoA, and gene activity is induced most efficiently by protocatechuate. Structural comparison of apo-PcaV and PcaV in complex with two molecules of protocatechuate revealed ligand binding at the deep hydrophobic pockets near the dimer interface (Fig. 1C). Residues in the dimer interface and residues in the wHTH motif separately superpose well (with RMSDs of ~ 0.4 Å) between the apo- and ligand-bound structures; however, a rigid body movement of the wHTH domain is induced on ligand binding in which the wHTH motif rotates up towards the dimer interface by ~ 15°, a conformation in which recognition helices would be unable to bind consecutive DNA major grooves [28].

Taken together, structural analyses of these transcription factors that all bind similar ligands and control related catabolic pathways illustrate three distinct mechanisms by which ligand-binding may prevent the protein from associating with cognate DNA and repressing transcription; 1 — by a conformational selection in which ligand binds and stabilizes a conformation of the apo-protein that is incompatible with DNA binding, 2 — by inducing conformational changes in the DNA-compatible apo-protein that preclude DNA binding, or 3 — by steric occlusion of DNA-binding surfaces.

### 3.2 Regulation of Anabolic Pathways

In all cases illustrated above, the MarR protein may be perceived as an on-off switch in which ligand is the trigger. This is congruent with the need to turn on catabolic genes only when the relevant substrate is present. However, other proteins have been described that may be considered more like a dimmer-switch that fine-tunes expression of genes in their regulon. In *Streptococcus pneumoniae*, the energetically expensive fatty acid biosynthesis is tightly regulated by several transcription factors, including FabT, which represses expression of *fab* genes. FabT associates with long-chain acyl-ACP, the small acyl carrier protein in which fatty acids are esterified to its phosphopantetheine prosthetic group. The acyl-ACP confers on FabT a higher affinity for cognate DNA sites, thereby enhancing repression under conditions of fatty acid sufficiency [29].

Most of the characterized MarR proteins function as repressors, however, activators have been reported as well. For instance, synthesis of the sesquiterpene antibiotic pentalenolactone in *Streptomyces exfoliatus* UC5319 is under control of PenR. PenR activates expression of the biosynthetic genes, and it is displaced from DNA by binding pentalenolactone or late-stage biosynthetic intermediates; this feed-back mechanism allows transcription to be reduced upon accumulation of the final product of the biosynthetic pathway [30].

### 3.3 Control of Metabolic Pathways in Response to Stress

Metabolic pathways are frequently regulated in response to stress, as exemplified by the wide-spread repression of gene activity during the bacterial stringent response, when genes associated with growth are repressed in favor of genes linked to survival [31]. In *S. coelicolor*, TamR (*trans*-aconitate methyltransferase regulator) represses multiple genes linked to the citric acid cycle, including the gene encoding aconitase [32], [33]; the [4Fe–4S] iron–sulfur cluster-containing enzyme aconitase catalyzes the isomerization of citrate to isocitrate via *cis*-aconitate. Citrate and *trans*-aconitate levels may increase when the iron-sulfur cluster required for enzymatic activity is damaged by reactive oxygen species, causing accumulation of the substrate citrate and release of the intermediate *cis*-aconitate, which is then converted to the more stable *trans* isomer [34]. These metabolites in turn bind TamR to attenuate DNA binding and relieve repression of cognate genes [32], [33]. Thus, TamR is another example of a MarR protein that functions as a dimmer-switch to fine-tune metabolic flux through the citric acid cycle, particularly during recovery from oxidative stress.

## 4 Exploitation of Transcription Factors for Metabolic Engineering and Biosensor Design

A thorough understanding of transcriptional regulatory networks responsible for optimizing cellular metabolism is essential, not only from the perspective of bacterial physiology but for optimization of such networks for industrial applications or for generation of biosensors. The interest in metabolic engineering in which existing metabolic fluxes are redirected to target pathways or heterologous pathways are expressed is fueled by prospects for commercial generation of value-added compounds such as biofuels and polymer precursors and by switching to sustainable “green” production. Metabolic engineering of bacteria to produce desirable products is challenging, however, as linked reactions may be adversely affected by channeling intermediates towards a specific pathway, resulting in a failure to maintain metabolic balance and leading to a reduced yield of the desired compound [35]. Toxicity of products or pathway intermediates may also hamper development of productive microbial factories. Dynamic regulation of target pathways combined with modeling of metabolic networks is therefore essential to maintain metabolic homeostasis. Such dynamic regulation may be accomplished by cellular biosensors that detect either environmental signals or cellular metabolites and produce a predetermined outcome. Some metabolite-sensing transcription factors have been successfully integrated into synthetic regulatory circuits enabling detection of a variety of compounds [36].

### 4.1 Biofuels

Hydrolysis of plant biomass is actively considered for generation of renewable energy such as biofuels and for production of aromatic precursors in plastics manufacturing [37], [38], [39]. However, aromatic compounds deriving from lignin degradation have proven inhibitory to the fermentation of glucose released from cellulose, and mechanisms for their removal or optimized degradation are therefore required [40]. Conversion of lignin into usable aromatics is likewise challenging [39]. The initial steps in bacterial lignin degradation remain incompletely understood, but downstream processing of initial products occurs via pathways for aromatic compound degradation that are well characterized, such as the β-ketoadipate and protochatechuate pathways discussed above. Engineering of biosensors based on transcription factors that respond to aromatic compounds derived from lignin degradation may be envisioned in which their cognate DNA sites are built into promoters driving expression of both catabolic enzymes as well as genes encoding accessory proteins required to maintain metabolic balance and/or export of potentially toxic compounds.

### 4.2 Biological Sensors: A Case Study of Urate Detection

Synthetic biology, the (re)design and implementation of novel biological devices or circuits, aims to create systems with predictable function. Transcription factors play a major role in biosensor design, since they can be implemented in synthetic circuits controlling gene expression in response to specific ligands. Such synthetic circuits may be designed to control downstream metabolic functions in response to changes in the environment or an imbalance of cellular metabolites. Such circuits have been successfully designed based on bacterial transcription factors with specificity for cellular metabolites. However, redesign of transcription factors to respond to new effector molecules remains a challenge, as changes in the ligand-binding residues required to accommodate novel scaffolds may disrupt allosteric communication with the DNA-binding domain [41], [42].

Synthetic sensor–effector gene networks may be designed to correct metabolic defects and restore metabolic homeostasis. One successful application of a ligand-responsive transcription factor for this purpose was aimed at treating tumor lysis syndrome or gouty arthritis by sensing and reducing levels of urate in the bloodstream [43], [44]. The sensor protein was *Deinococcus radiodurans* HucR. In *D. radiodurans*, the gene encoding uricase is divergently oriented from *hucR*; HucR binds urate with low micromolar affinity, a binding event that leads to loss of DNA binding and upregulation of the divergently oriented genes. HucR binds with high affinity and specificity to a single DNA site in the intergenic region spanning genes encoding uricase and HucR [45], [46]. These properties of HucR make it an ideal sensor of urate.

A synthetic mammalian circuit designed to maintain urate homeostasis in the bloodstream was created in which detection of urate by HucR results in derepression of a gene encoding *Apergillus flavus* uricase, and microencapsulated cells engineered to express this prosthetic gene network successfully reduced serum levels of urate in mice [43]. HucR was also used to create a device in which uricase was incorporated into a polyacrylamide hydrogel that was crosslinked by HucR binding to its cognate DNA site; application of urate in turn releases the encapsulated uricase [44]. More recently, HucR was also integral to development of a urate biosensor in which presence of urate is translated into a luminescent signal [47]. Since HucR also is sensitive to changes in pH, it will be interesting to ascertain its utility in synthetic devices that depend on detection of pH changes [13].

HucR binds urate in preference to xanthine, a preference ascribed to conformational changes induced only on binding of a negatively charged ligand [46]. Thus, HucR may not be the sensor of choice for the detection of xanthine that is important both in food industries and for clinical diagnosis of xanthinuria [48]. While the development of xanthine biosensors based on immobilized xanthine oxidase enzyme has been reported, nature's “redesign” of HucR may offer a viable alternative: *Agrobacterium fabrum* encodes a homolog of HucR, named PecS, which binds urate and xanthine with equivalent low-micromolar affinity, and both ligands effectively attenuate the specific binding of PecS to its cognate DNA site [49].

## 5 Summary and Outlook

As exemplified above, MarR family transcription factors frequently bind phenolic compounds. This property makes them ideally suited for regulation of genes encoding enzymes involved, for example, in degradation of lignin-derived aromatics as well as man-made environmental pollutants [8]. A comparison of proposed mechanisms by which ligand binding leads to altered DNA binding also makes it clear that there is not a unique mechanism by which ligand-binding imposes a change in DNA binding. While many MarR proteins share a ligand-binding “hot-spot” in a deep crevice between dimerization and DNA-binding regions of the protein, specific outcomes of ligand binding vary and must be determined on a case-by-case basis.

The ability to sense metabolites as a readout of cellular metabolic state makes MarR proteins well suited for the development of biosensors. So far, such detection has relied on the native ligand-specificity, as illustrated by the successful implementation of HucR in several urate-sensing devices. Because these transcription factors are very sensitive to changes that alter the dynamics of the dimer interface, redesign of ligand-specificity may be confounded by resulting changes in communication between ligand-binding pockets and the DNA-binding wHTH motif. While identification of cognate DNA sites for MarR proteins is frequently facile due to the presence of palindromic sequences in *marR* gene promoters, identification of specific ligands may be a challenge. Expanding the inventory of MarR proteins with known DNA- and ligand-specificities promises not only to further understanding of bacterial metabolism, but also to develop a larger repertoire of biosensors with clinical potential.
